# Multivariate Analysis of the Spatial Distance Between the Corneal Thinnest Point and Maximum Keratometry in Keratoconic Corneas: Association with Disease Severity

**DOI:** 10.3390/diagnostics16193194

**Published:** 2026-10-01

**Authors:** Umberto Camellin, Marco Lombardo, Francesco Franchina, Ivan Ninotta, Gianluigi Latino, Antonio Valastro, Pasquale Aragona, Domenico Schiano-Lomoriello, Anna M. Roszkowska

**Affiliations:** 1 Department of Biomedical and Dental Sciences and Morphofunctional Imaging, University of Messina, 98125 Messina, Italy; umberto.camellin.uc@gmail.com (U.C.);; 2 Studio Italiano di Oftalmologia, Via Livenza 3, 00198 Rome, Italy; 3 Vision Engineering Italy srl, Via Livenza 3, 00198 Rome, Italy; 4 UniCamillus-Saint Camillus International University of Health Sciences, 00131 Rome, Italy; 5 IRCCS Fondazione G.B. Bietti, 00198 Rome, Italy; 6 Faculty of Medicine, Andrzej Frycz Modrzewski Krakow University, 30-705 Krakow, Poland

**Keywords:** thinnest point, keratoconus, maximum keratometry

## Abstract

**Background:** This study quantified the planar distance (D, mm) between the corneal thinnest point (TP) and maximum keratometry (Kmax) and examined its association with tomographic features and keratoconus severity. **Methods:** Tomographic data from 140 patients (280 eyes) with bilateral manifest keratoconus were obtained with a Scheimpflug camera (Pentacam HR; Oculus, Wetzlar, Germany). D was calculated from the Cartesian coordinates of TP and Kmax. A multiple linear regression model included simulated anterior average keratometry (SimKavg), anterior and posterior radii of curvature (ARC and PRC), and anterior Q-value (Q-ant). Inference used patient-clustered robust standard errors. Internal validation comprised repeated grouped 10-fold cross-validation and patient-level bootstrap resampling. Performance was assessed using R^2^, root-mean-square error (RMSE), mean absolute error (MAE), and Bland–Altman limits of agreement. **Results:** The model explained 51.8% of the apparent variance in D (adjusted R^2^ = 0.511; *p* < 0.001). Apparent RMSE and MAE were 0.568 and 0.428 mm. Mean grouped cross-validated performance was R^2^ = 0.472, RMSE = 0.594 mm, and MAE = 0.443 mm. The out-of-fold bias was −0.005 mm, with 95% limits of agreement from −1.162 to +1.151 mm. ARC, SimKavg, and Q-ant remained significant with patient-clustered inference, whereas PRC did not (*p* = 0.111). **Conclusions:** D was associated with keratoconus severity and could be estimated with moderate internal performance from routinely available tomographic variables. Collinearity, fellow-eye dependence, and limits of agreement of approximately ±1.1 mm restrict interpretation of individual coefficients and preclude interchangeability with direct measurement. External and longitudinal validation is required before clinical use.

## 1. Introduction

Keratoconus (KC) is a bilateral, asymmetric corneal ectasia characterized by progressive stromal thinning and steepening, potentially leading to marked visual impairment. Its reported prevalence varies with geographic setting, ethnicity, age, and diagnostic method; an Italian population-based estimate has also shown that KC is more frequent than historically assumed [1,2]. KC commonly manifests in adolescence or early adulthood and may progress for several decades [1,2,3,4,5].

Corneal cross-linking (CXL) is an established procedure in the management of progressive KC [6,7]. The procedure aims to increase tissue resistance and stiffness by generating additional covalent bonds between collagen fibrils and proteoglycan core proteins through a riboflavin–ultraviolet A photochemical reaction, thereby slowing or arresting ectatic progression [8,9]. Long-term clinical follow-up has shown stabilization after CXL, and its introduction has been associated with a reduction in keratoplasty procedures, indirectly supporting sustained efficacy in preventing progression [10].

Early detection of keratoconus is important because progressive stromal thinning and asymmetric steepening increase corneal conicity and irregularity and may ultimately produce irreversible visual and structural deterioration. Several diagnostic instruments are available, and corneal tomography provides concurrent assessment of anterior and posterior surfaces and corneal thickness [11,12,13,14,15,16,17]. Single-point indices such as Kmax and TP capture different manifestations of ectasia but do not describe whether focal steepening and focal thinning are spatially separated or convergent [18,19,20,21]. Because no individual index captures all structural manifestations of progression, concordant change across multiple tomographic domains is generally more informative than an isolated single-point change. The ABCD system provides a multidimensional description based on anterior and posterior radii of curvature, thinnest pachymetry, and corrected distance visual acuity [17]. Its progression display can support component-wise serial assessment before and after treatment, although the present cross-sectional study did not evaluate treatment response. The ABCD system is used here for severity characterization; it is not treated as an unqualified reference standard or as evidence that D is a validated progression marker.

The aim of the present study was to quantify the planar distance between TP and Kmax in keratoconic eyes, investigate its association with routinely available tomographic variables and severity, and evaluate the internal performance of a regression equation for estimating D. As a spatial descriptor, D may complement the magnitude-based components of the ABCD system by indicating whether focal thinning and maximal steepening remain separated or become more closely aligned. The study was not designed to establish D as a diagnostic discriminator, longitudinal progression biomarker, or treatment-response endpoint, and any value for monitoring CXL response requires prospective evaluation.

## 2. Materials and Methods

This retrospective observational study included patients with a confirmed diagnosis of bilateral manifest keratoconus followed at the Corneal and Refractive Surgery Segment of the Ophthalmology Unit of the University Hospital of Messina. To obtain a tomographically homogeneous untreated cohort and minimise potential effects on curvature, ocular surface, fixation, or scan quality, eyes with corneal opacity, previous corneal infection, or any prior ocular or corneal surgery—including CXL, laser refractive surgery, or combined protocols—were excluded. Written informed consent was obtained before examination. The Institutional Ethics Committee approved the study (protocol no. 0013263/2022), which was conducted in accordance with the Declaration of Helsinki (2013 revision).

All patients discontinued soft and rigid gas-permeable contact lenses for at least 2 and 4 weeks, respectively. Examination included visual acuity and slit-lamp biomicroscopy. Tomography was performed with a Scheimpflug camera (Pentacam HR; Oculus, Wetzlar, Germany). One experienced operator acquired three consecutive scans per eye, and their mean was used for analysis. Two corneal specialists independently reviewed the examinations; only datasets for which both specialists agreed on manifest keratoconus and scan suitability were retained. No single severity classification served as the diagnostic inclusion rule. Diagnosis was based on concordant clinical and tomographic evidence, including anterior and posterior curvature abnormalities, focal pachymetric thinning, and asymmetry indices. Amsler–Krumeich and ABCD were subsequently used to characterize severity. Recorded variables included Kmax, TP, and their Cartesian coordinates (TPx, TPy, Kmax-x, and Kmax-y) relative to the device-defined tomographic centre. Coordinates were taken directly from the Pentacam software (1.26r28) output and were not independently reconstructed from raw elevation or curvature data.

Additional tomographic parameters included the simulated anterior keratometry (SimKavg), mean posterior keratometry (Kpostavg), anterior asphericity (Q-value at 8 mm; Q-ant) and posterior asphericity (Q-value at 8 mm; Q-post). The anterior radius of curvature (ARC) and posterior radius of curvature (PRC) within the 3.0-mm zone centered on the corneal thinnest point, corresponding to parameters A and B of the Belin ABCD system, respectively, were also collected from the Belin ABCD Progression Display of the Pentacam device [17].

### 2.1. Theoretical Calculation and Development of Regression Formula

The distance (D, mm) between the TP and K_max_ points was calculated using the following trigonometric formula:

(1) $$D\;=\sqrt{{\left(Kmax\left(x\right)-TP(x)\right)}^{2}+{\left(Kmax\left(y\right)-TP(y)\right)}^{2}}$$

The relationship between the parameter D (mm) and SimK_avg_, Kpost_avg_, ARC, PRC, Q-ant, and Q-post was analyzed.

### 2.2. Statistical Analysis

Distributional assumptions were assessed using the Kolmogorov–Smirnov test and inspection of the data. Paired normally distributed measurements were compared with paired *t*-tests; paired non-normally distributed measurements were compared with the Wilcoxon signed-rank test. Pearson or Spearman correlation coefficients were used as appropriate. All tests were two-sided, with *p* < 0.05 considered statistically significant.

The planar TP–Kmax distance was calculated as D = √[(TPx − Kmax-x)^2^ + (TPy − Kmax-y)^2^]. A multiple linear regression model was fitted with D as the dependent variable and ARC, PRC, SimKavg, and Q-ant as predictors. These variables correspond to the final model retained from the initial candidate set, which also included Kpostavg and Q-post. Multicollinearity was evaluated using variance inflation factors (VIFs), tolerance, and the condition index.

Because both eyes were available for every participant, coefficient inference for the 280-eye regression used patient-clustered robust standard errors. Internal validation used 100 repetitions of grouped 10-fold cross-validation, with both eyes from a participant assigned to the same fold, and 1000 patient-level bootstrap resamples. Performance measures were R^2^, RMSE, MAE, and Bland–Altman bias and 95% limits of agreement. Sensitivity analyses included separate right- and left-eye models and a model using one randomly selected eye per participant. Sex and ABCD-stage analyses were considered exploratory.

As this was a retrospective study, no prospective sample-size calculation was performed. A post hoc adequacy assessment for a four-predictor regression, assuming a medium effect size (f^2^ = 0.15), α = 0.05, and 80% power, indicated that approximately 85 independent participants would be required; the study included 140 participants.

Initial data management and conventional analyses were performed using IBM SPSS Statistics version 23.0. Clustered inference, internal validation, Bland–Altman analysis, and sensitivity analyses were performed in R version 4.5.2.

## 3. Results

**Figure 1:**
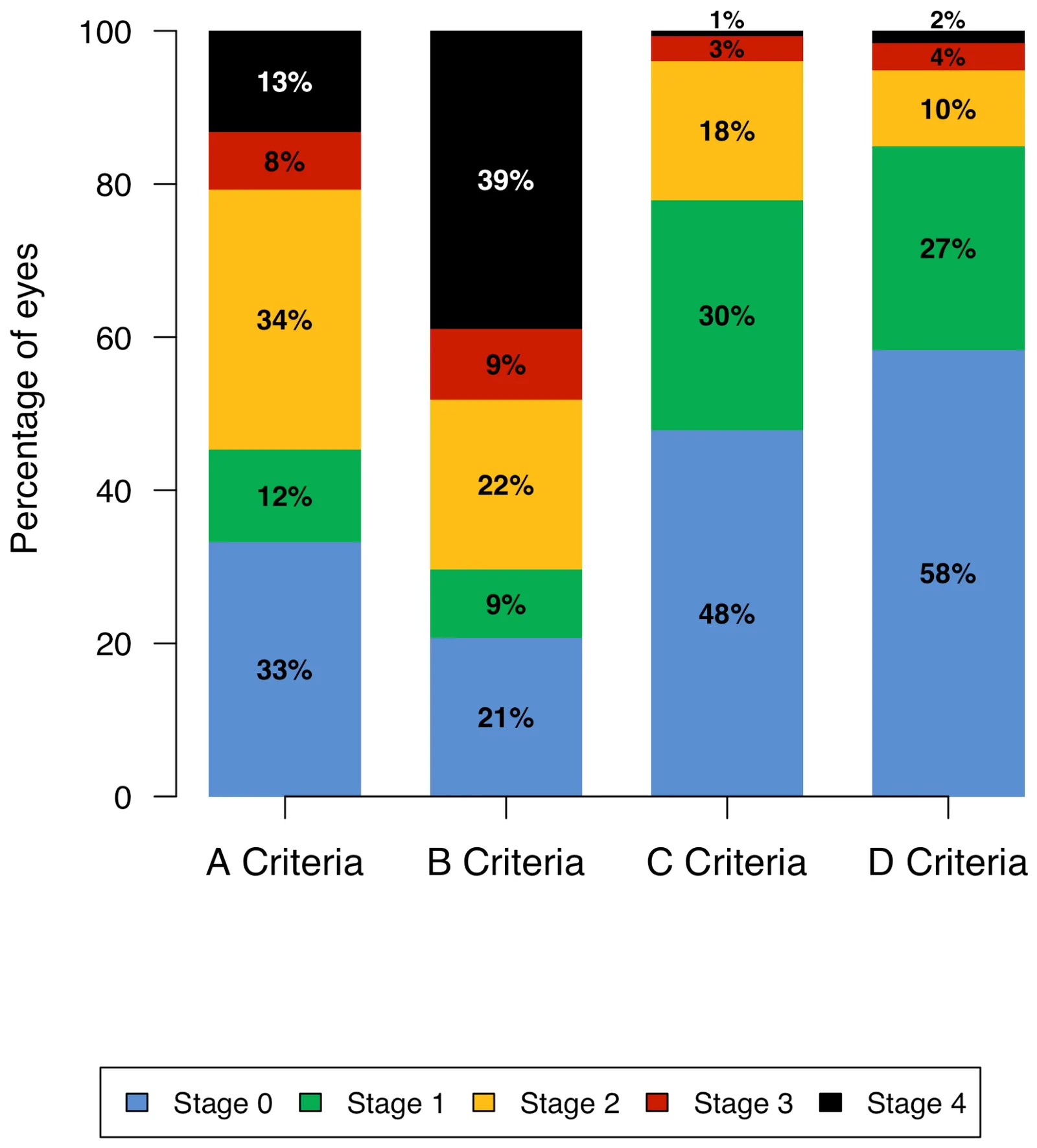
Distribution of eyes according to the staging values of each parameter of the Belin ABCD system. (**A**) Anterior radius of curvature in the 3.0-mm zone centred on the thinnest corneal location (ARC); (**B**) posterior radius of curvature in the 3.0-mm zone centred on the thinnest corneal location (PRC); (**C**) thinnest pachymetry (TP; μm); (**D**) corrected distance visual acuity (CDVA). Parameter B was most frequently classified as stage 4. Because the ABCD components represent different anatomical or functional domains and have unequal category sizes, their distributions should not be interpreted as evidence that one component necessarily changes earlier than another.

A total of 280 eyes of 140 patients (89 males and 51 females) with bilateral, manifest, keratoconus were included in the study. The age ranged from 11 to 69 years (average 35.7 ± 13.4 years). According to the Amsler-Krumeich classification, the study population included 221 eyes (79%) with grade 1, 45 eyes (16%) with grade 2, 8 eyes (3%) with grade 3, and 6 eyes (2%) with grade 4 keratoconus. Keratoconus severity distribution according to the ABCD classification is shown in Figure 1.

Corneal tomographic data are reported in Table 1.

In the right eye (Table 1), the mean (±SD) TP_x_ and K_max-x_ values were −0.57 ± 0.28 mm and +0.14 ± 0.66 mm (*p* < 0.05) respectively and the mean (±SD) TP_y_ and K_max-y_ values were −0.68 ± 0.34 mm and −1.41 ± 1.15 mm (*p* < 0.05) respectively. In the left eye (Table 1), the mean (±SD) TP_x_ and K_max-x_ values were +0.50 ± 0.37 mm and −0.08 ± 0.60 mm (*p* < 0.05) respectively and the mean (±SD) TP_y_ and K_max-y_ values were −0.76 ± 0.37 mm and −1.36 ± 1.20 mm (*p* < 0.05) respectively. Spearman correlation analysis between anterior and posterior tomographic parameters in right and left eyes is shown in Table 2. A highly significant correlation was observed for all analyzed variables, including SimK_avg_, K_post_, Q-ant, and Q-post. Kmax-x and Kmax-y values, which showed a normal distribution, were compared with ARC and PRC using Pearson’s correlation coefficient.

No significant correlation was found between either Kmax coordinate component and the corresponding curvature parameters (R^2^ = 0.009 for ARC and R^2^ = 0.010 for PRC). Table 3 reports the coordinate correlations. The vertical TPy–Kmax-y association was r = 0.460 in right eyes and r = 0.591 in left eyes, compared with TPx–Kmax-x correlations of r = 0.270 and r = 0.165, respectively.

**Figure 2:**
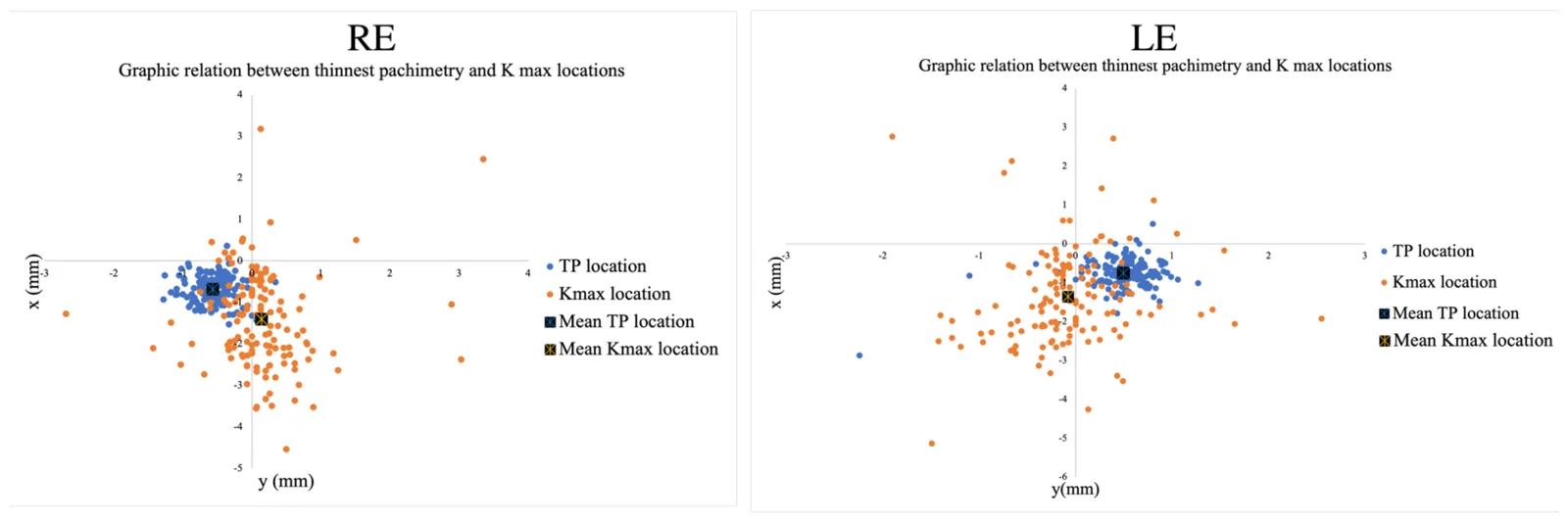
Cartesian distribution of the thinnest point pachymetry (TP) and maximum curvature (K_max_) locations relative to the corneal tomography center [x(0), y(0)] in fellow eyes of the study population. Compared with TP, K_max_ locations showed a wider spatial dispersion. Overall, the TP location was consistently displaced temporally and superiorly relative to the corresponding K_max_ location. Mirror symmetry (enantiomorphism) is evident when corresponding locations from fellow eyes are compared.

As shown in Figure 2, the TP was systematically located temporally and superiorly relative to the K_max_ position in both eyes. The mean inter-point distance between K_max_ and TP locations (D parameter) in the study population was 1.36 ± 0.82 mm.

The multiple linear regression model including SimKavg, ARC, PRC, and Q-ant was significant (R^2^ = 0.518; adjusted R^2^ = 0.511; F [4275] = 74.02; *p* < 0.001). The fitted equation was D = −7.803 + 0.898 × ARC − 0.368 × PRC + 0.124 × SimKavg + 1.527 × Q-ant. Apparent RMSE and MAE were 0.568 and 0.428 mm, respectively. VIF values were 11.49 for ARC, 9.33 for PRC, 3.88 for SimKavg, and 2.41 for Q-ant; corresponding tolerance values were 0.087, 0.107, 0.258, and 0.415. The maximum condition index was 8.08, indicating relevant collinearity, particularly for ARC and PRC.

In univariable analyses, larger ARC and PRC values were associated with greater spatial separation between TP and Kmax (ARC: r = 0.507, *p* = 0.023; PRC: r = 0.467, *p* = 0.002), whereas greater anterior and posterior keratometric steepness was associated with shorter D (r = −0.395 and r = −0.416, respectively; *p* = 0.017). Q-ant also correlated with D (r = 0.645; *p* < 0.001). These marginal associations should be distinguished from the adjusted regression coefficients because ARC, PRC, and keratometric measures were correlated. D decreased across increasing stages of the individual ABCD components (all Kruskal–Wallis *p* < 0.001; Supplementary Table S1), although the most advanced C and D categories contained few observations.

Based on multiple linear regression analysis, the following predictive model (Equation (2)) was derived:

(2) $$D\left(mm\right)=-7.803+0.898*ARC-0.368*PRC+0.124*SimKavg+1.527*Qant$$

**Figure 3:**
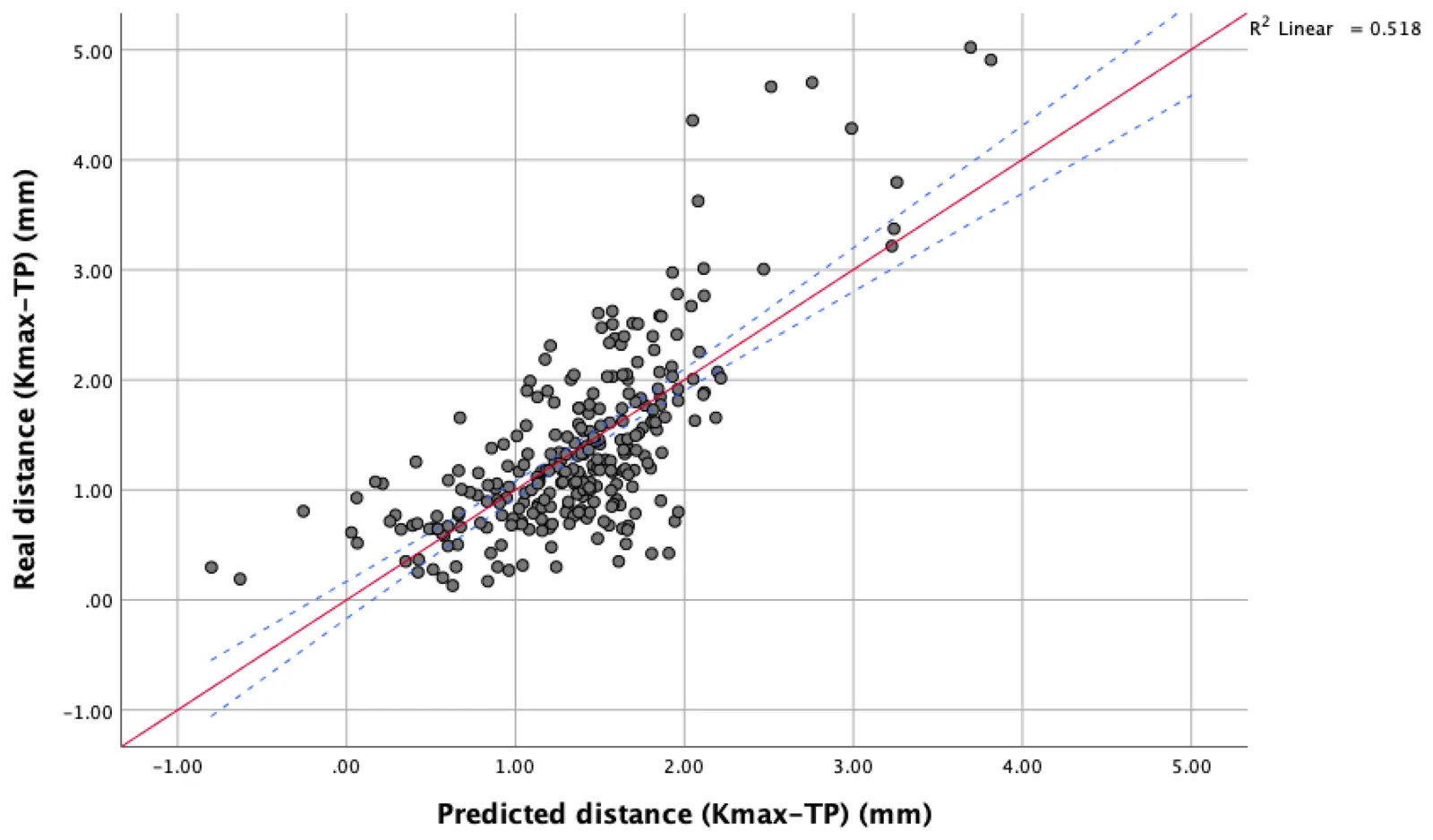
Relationship between fitted and measured TP–Kmax distance (D, mm) in the 280 study eyes. The continuous line represents the regression fit and the curved dotted lines represent its 95% confidence interval. Individual predictive agreement was evaluated separately using grouped out-of-fold RMSE, MAE, and Bland–Altman limits of agreement.

The mean fitted distance was 1.36 ± 0.59 mm. Apparent predicted-minus-observed bias was approximately 0.000 mm, with 95% limits of agreement from −1.114 to +1.114 mm. In grouped repeated 10-fold cross-validation, mean R^2^ was 0.472, RMSE was 0.594 mm, and MAE was 0.443 mm. Out-of-fold bias was −0.005 mm, with limits of agreement from −1.162 to +1.151 mm. Patient-level bootstrap validation yielded an optimism-corrected R^2^ of 0.492 and RMSE of 0.591 mm. Figure 3 shows the relationship between fitted and measured D; individual prediction error is summarized by the cross-validated metrics and limits of agreement.

With patient-clustered robust standard errors, ARC (β = 0.898, SE = 0.309, *p* = 0.004), SimKavg (β = 0.124, SE = 0.026, *p* < 0.001), and Q-ant (β = 1.527, SE = 0.164, *p* < 0.001) remained significant, whereas PRC did not (β = −0.368, SE = 0.229, *p* = 0.111). In the one-eye-per-participant sensitivity analysis, overall performance was similar (R^2^ = 0.544), while PRC again did not reach statistical significance (*p* = 0.074). Right- and left-eye models yielded R^2^ values of 0.557 and 0.532, respectively.

The cohort comprised 51 women and 89 men. At participant level, mean D was 1.288 ± 0.657 mm in women and 1.402 ± 0.709 mm in men; the unadjusted comparison was not significant (*p* = 0.339). In an exploratory adjusted model, male sex was associated with a 0.196-mm greater D (patient-clustered SE = 0.082; *p* = 0.018). Because this analysis was not prespecified and crude and adjusted findings differed, it was not used to modify the primary conclusions (Supplementary Table S2).

## 4. Discussion

This study quantified the planar distance between TP and Kmax and examined its tomographic correlates in 280 eyes of 140 patients. TP was generally temporal and superior to Kmax, whereas Kmax showed wider spatial dispersion. The four-variable equation explained approximately half of the apparent variance in D and retained moderate performance after grouped internal validation. However, collinearity and patient-clustered inference materially affected the interpretation of individual coefficients: ARC, SimKavg, and Q-ant remained significant, whereas the contribution of PRC was not robust after fellow-eye dependence was considered.

Overall, the present findings are consistent with those of Tian et al. [15], who analyzed the Cartesian distance between the TP and Kmax and similarly reported a progressive decrease in the D parameter with increasing keratoconus severity. Specifically, the mean D value was 1.04 mm in healthy eyes (control group), 0.98 mm in stage I keratoconus, and 0.43 mm in stage IV keratoconus. Unlike the study by Tian et al., however, the present study identified the corneal tomographic variables associated with the D parameter and developed a multivariable predictive model based on SimKavg, ARC, PRC and Q-ant, allowing estimation of the TP–Kmax distance from routinely available tomographic parameters.

Similar findings for the D parameter were reported by Kaya et al. [22] and Ashwin et al. [23,24,25,26].

Specifically, Ashwin et al. found an average value of 0.70 mm from the tomographic center, as measured by the Pentacam device, in healthy eyes. However, the authors did not investigate the relationship between this parameter and other corneal tomography variables [25]. Kaya et al. reported a significant increase in the distance between the TP position and the tomographic center, as measured with the Orbscan II device, in eyes with keratoconus. Furthermore, this distance significantly was associated with disease stage [22].

The main contribution of the study is the quantitative assessment of how routinely available tomographic variables relate to the TP–Kmax offset. The equation should not be interpreted as a substitute for direct Pentacam measurement: cross-validated limits of agreement of approximately ±1.1 mm indicate potentially relevant individual error. Likewise, the cross-sectional design does not establish that serial change in D detects progression or improves CXL decisions. The findings instead provide a basis for prospective studies of repeatability, minimal detectable change, and longitudinal prognostic value.

The observed relationships may reflect both ectatic geometry and the way tomography is represented. Global curvature affects the relationship between chordwise and surface distance: for the same projected separation, a steeper or more prolate cornea may have a different surface arc length and sagittal separation. D is a planar Euclidean distance between software-derived Cartesian coordinates, not a geodesic distance along the three-dimensional corneal surface. Corneal prolateness, variable sagittal depth, reference-axis decentration, and angle kappa may therefore influence the apparent spatial offset. Refractive status may also be associated with ocular geometry and fixation behaviour; axial length, anterior chamber depth, pupil size or decentration, and the relationship between the visual, pupillary, and tomographic axes can affect centre-referenced landmark locations. These biometric factors were not measured in this study. In addition, TP and Kmax were exported after proprietary smoothing, fitting, and interpolation; their coordinates were not independently verified from raw data.

In this regard, Velázquez et al. [27] performed a morphometric analysis of 440 keratoconic eyes using a three-dimensional characterization of the cornea rather than conventional two-dimensional topographic maps. Their three-dimensional tomographic models demonstrated higher diagnostic accuracy than two-dimensional approaches. Therefore, the progressive shortening of the D parameter observed in the present study with increasing keratoconus severity may, at least in part, reflect the intrinsic limitations of two-dimensional tomographic representations rather than the actual three-dimensional spatial relationship between the TP and Kmax.

According to our results, an increase in anterior corneal curvature corresponds to an increase in posterior corneal curvature, which is associated with an increase in corneal asphericity (Table 2) [28,29]. A possible explanation for apparently limited early anterior-surface change is compensatory epithelial remodelling: epithelial thinning over the cone and relative thickening around it may partially smooth stromal irregularity on the anterior topographic surface. Because epithelial thickness was not measured, this mechanism remains interpretative rather than demonstrated by the present data.

These findings are consistent with previous studies, which have shown that the posterior-to-anterior curvature ratio is significantly lower in eyes with keratoconus than in healthy eyes. Accordingly, simultaneous assessment of anterior and posterior corneal curvature has been proposed as a valuable approach for the detection and monitoring of keratoconus progression [13].

The established observation that the TP and Kmax do not spatially coincide has prompted the development of alternative approaches to conventional methodologies based on a single tomographic index (i.e., Kmax, TP, etc.) for monitoring keratoconus progression. Lombardo et al. [19]. proposed a 3-mm zonal analysis of corneal tomography centered 1.5 mm inferior to the videokeratographic center and demonstrated that this approach provided a higher accuracy than single-point indices (TP and Kmax) for detecting keratoconus progression over a 4-year follow-up period. The zonal analysis was able to capture regional changes in corneal curvature and thickness within the area of interest, which could not be adequately characterized by Kmax or TP alone [19,20,21].

The vertical association between TP and Kmax coordinates was stronger than the horizontal association, particularly in left eyes (Table 3). This may relate to the preferential inferior expression of ectasia and asymmetric stromal architecture [30,31]. Vector analysis was not performed and remains a potential direction for future work. The finding should not be taken to show that D adds diagnostic or prognostic information beyond established tomography and biomechanical assessment [32,33].

Recent studies published between 2024 and 2026 provide complementary context but did not evaluate the same TP-Kmax distance. Vilares-Morgado et al. longitudinally assessed Kmax, minimum pachymetry, PRC, and the Belin/Ambrosio D-index after transepithelial CXL; Hammoud et al. measured the distance between the thinnest point and focal biomechanical weakening using motion-tracking Brillouin microscopy; and Villa-Collar et al. demonstrated limited agreement among severity classifications based on anterior topographic parameters [34,35,36]. These studies reinforce the value of multidimensional and spatial assessment while also showing that their endpoints are not directly interchangeable with the planar D examined here. A structured comparison is provided in Supplementary Table S3.

## 5. Limitation

Several limitations require consideration. The retrospective single-centre design creates potential selection bias, and the absence of protocol-matched healthy controls prevents assessment of disease specificity or KC-versus-normal discrimination. Severity was imbalanced, reducing precision in advanced categories. Both eyes were included, although clustered inference and one-eye sensitivity analysis were used. ARC and PRC showed relevant collinearity, which limits interpretation of individual coefficients. Posterior-surface variables may be more sensitive to scan quality and irregularity, particularly in more advanced KC [37]. Coordinates were device-derived and dependent on proprietary algorithms, and D was planar rather than truly three-dimensional. Parameter D of the ABCD system was unavailable in 88 eyes. Finally, internal validation does not replace external validation, and no longitudinal progression or treatment-response data were available.

## 6. Conclusions

The TP–Kmax distance was associated with keratoconus severity and could be estimated with moderate internal performance using routinely available tomographic variables. Nevertheless, relevant collinearity, fellow-eye dependence, and limits of agreement of approximately ±1.1 mm restrict interpretation of the individual coefficients and preclude interchangeability with direct measurement. External and longitudinal validation is required before D or its predictive equation can be used to monitor progression or guide treatment decisions.

## Figures and Tables

**Table 1:** Corneal tomographic data in study population.

	SimK_avg_ (D)	Q-Ant Value	Kpost_avg_ (D)	Q-Post Value	CCT (μm)	TP (μm)	TP_x_ (mm)	TP_y_ (mm)	K_max_ (D)	K_max-x_ (mm)	K_max-y_ (mm)
**All eyes (280 eyes)**											
**Mean**	46.26	−0.58	−6.77	−0.55	501	485	−0.04	−0.72	52.81	0.03	−1.38
**SD**	3.75	0.42	0.75	0.43	47	48	0.62	0.36	7.57	0.64	1.18
**Min.**	40	−2	−10	−2	322	299	−2	−3	42	−3	−5
**Max.**	62	1	−6	1	659	619	1	1	98	3	3
**Right Eye (*n* = 140)**											
**Mean**	46.18	−0.56	−6.75	−0.55	504	489	−0.57	−0.68	52.64	0.14	−1.41
**SD**	3.64	0.42	0.71	0.41	45	48	0.28	0.34	7.40	0.66	1.15
**Min.**	40.20	−2.24	−9.70	−1.67	331	299	−1.28	−1.53	42.60	−2.69	−4.54
**Max.**	61.50	0.86	−5.50	0.51	623	619	0.34	0.36	87.40	3.35	3.18
**Left Eye (*n* = 140)**											
**Mean**	46.33	−0.59	−6.78	−0.56	498	480	0.50	−0.76	52.98	−0.08	−1.36
**SD**	3.86	0.43	0.80	0.45	48	48	0.37	0.37	7.76	0.60	1.20
**Min.**	40.20	−2.44	−10.10	−1.82	322	299	−2.24	−2.87	41.50	−1.90	−5.14
**Max.**	62.15	0.46	−5.55	0.70	659	618	1.27	0.51	98.20	2.55	2.76

CCT, central corneal thickness; K_max_, maximum keratometry index; K_max-x and y_, cartesian distances of K_max_ from tomographic center; K_post_, posterior keratometry; SimK_avg_, average simulated keratometry; TP, thinnest point pachymetry; TP_x and y_, cartesian distances in mm of thinnest point location (TP_x_, TP_y_) from tomographic center; Q-ant value, anterior asphericity at 8 mm; Q-post value, posterior asphericity at 8 mm; Max., maximum values; Min., minimum values; SD, standard deviation; D, diopter; mm, millimeter; µm, micrometer.

**Table 2:** Spearman correlation analysis between anterior and posterior tomographic data in the right (*n* = 140) and left (*n* = 140) eyes of the study population.

	Right Eye			Left Eye		
	Kpost_avg_ (D)	Q-Ant	Q-Post	Kpost_avg_ (D)	Q-Ant	Q-Post
SimK_avg_ (D)	rs = −0.92, *p* < 0.05	rs = −0.67, *p* < 0.05	rs = −0.60, *p* < 0.05	rs = −0.93, *p* < 0.05	rs = −0.70, *p* < 0.05	rs = −0.60, *p* < 0.05
Kpost_avg_ (D)		rs = 0.73, *p* < 0.05	rs = 0.76, *p* < 0.05		rs = 0.75, *p* < 0.05	rs = 0.74, *p* < 0.05
Q-ant			rs = 0.85, *p* < 0.05			rs = 0.79, *p* < 0.05

K_post_, posterior keratometry; SimKavg, average simulated keratometry; Q-ant value, anterior asphericity at 8 mm; Q-post value, posterior asphericity at 8 mm.

**Table 3:** Correlation analysis between Cartesian coordinates (mm) of maximum points of curvature (Kmax-x, Kmax-y) and Cartesian coordinates (mm) of thinnest point locations (TPx, TPy) relative to the corneal tomographic centre.

	Right Eye		Left Eye	
	K_max-x_ (mm)	K_max-y_ (mm)	K_max-x_ (mm)	K_max-y_ (mm)
TP_x_ (mm)	r = 0.270, *p* < 0.05	r = 0.050, *p* = 0.56	r = 0.165, *p* = 0.52	r = −0.037, *p* = 0.66
TP_y_ (mm)	r = −0.132, *p* = 0.12	r = 0.460, *p* < 0.05	r = 0.297, *p* < 0.05	r = 0.591, *p* < 0.05

## Data Availability

The data supporting the findings of this study are available from the corresponding author upon reasonable request. The data are not publicly available due to privacy and ethical restrictions related to patient information.

## Supplementary Materials

The following supporting information can be downloaded at: https://www.mdpi.com/article/10.3390/diagnostics16193194/s1, Table S1: Distribution of the TP–Kmax distance (D) according to the individual ABCD components; Table S2: Exploratory analysis of the association between sex and TP–Kmax distance; Table S3: Comparison of recent studies evaluating spatial, tomographic, and biomechanical parameters in keratoconus.
