# Supplementary material for: Multivariate Analysis of the Spatial Distance Between the Corneal Thinnest Point and Maximum Keratometry in Keratoconic Corneas: Association with Disease Severity
Source: Diagnostics (Basel). 2026 Oct 1;16(19):3194. doi: 10.3390/diagnostics16193194 (PMC13664558; doi:10.3390/diagnostics16193194)
Supplement: Supplementary file 1 [file diagnostics-16-03194-s001.zip › diagnostics-4482664-supplementary.pdf]

SUPPLEMENTARY TABLES

Supplementary Table S1. TP–Kmax distance according to the stages of each Belin ABCD component.

Component	Stage	n	Mean ± SD (mm)	Median [Q1–Q3] (mm)
A	0	93	1.773 ± 0.995	1.626 [1.069–2.050]
A	1	34	1.550 ± 0.745	1.334 [0.881–2.236]
A	2	95	1.210 ± 0.555	1.162 [0.890–1.389]
A	3	21	1.033 ± 0.564	0.792 [0.650–1.329]
A	4	37	0.724 ± 0.413	0.692 [0.365–0.978]
B	0	58	1.941 ± 1.073	1.770 [1.247–2.362]
B	1	25	1.687 ± 0.950	1.457 [0.973–2.007]
B	2	62	1.417 ± 0.631	1.324 [0.956–1.739]
B	3	26	1.397 ± 0.630	1.185 [1.050–1.602]
B	4	109	0.937 ± 0.470	0.868 [0.645–1.181]
C	0	134	1.611 ± 0.907	1.435 [1.000–2.005]
C	1	84	1.201 ± 0.732	1.068 [0.763–1.354]
C	2	51	1.089 ± 0.524	1.001 [0.703–1.415]
C	3	9	0.861 ± 0.437	0.792 [0.645–1.166]
C	4	2	0.481 ± 0.186	0.481 [0.416–0.547]
D	0	112	1.525 ± 0.695	1.389 [1.050–1.871]
D	1	51	1.184 ± 0.805	0.978 [0.703–1.389]
D	2	19	1.003 ± 0.717	0.769 [0.574–1.218]
D	3	7	0.893 ± 0.255	0.792 [0.708–1.086]
D	4	3	1.277 ± 1.679	0.314 [0.307–1.765]

Kruskal–Wallis P<0.001 for each component. Exact frequencies are reported in the table; for example, A4 included 37/280 eyes (13.2%). Parameter D was unavailable in 88 eyes; percentages for D in Figure 1 use the 192 eyes with available CDVA staging. Advanced C and D categories should be interpreted cautiously because of small sample sizes.

Supplementary Table S2. Exploratory participant-level comparison by sex.

Variable	Women (n=51), mean ± SD	Men (n=89), mean ± SD	Women, median [IQR]	Men, median [IQR]	P value
D (mm)	1.288 ± 0.657	1.402 ± 0.709	1.111 [0.786–1.632]	1.310 [0.932–1.698]	0.339
ARC (mm)	6.936 ± 0.520	6.921 ± 0.595	7.025 [6.668–7.273]	6.965 [6.585–7.310]	0.694
PRC (mm)	5.231 ± 0.579	5.183 ± 0.609	5.270 [4.978–5.628]	5.210 [4.710–5.625]	0.651
SimKavg (D)	46.594 ± 3.038	46.061 ± 3.300	45.900 [44.438–48.213]	45.225 [43.950–47.350]	0.162
Q-ant	−0.569 ± 0.342	−0.579 ± 0.350	−0.510 [−0.763–−0.348]	−0.505 [−0.745–−0.365]	0.800

P values are from exploratory unadjusted participant-level comparisons. In the adjusted eye-level regression with patient-clustered standard errors, male sex was associated with a 0.196-mm greater D (P=0.018).

Supplementary Table S3. Comparison with recent studies published from 2024 to 2026.

Study	Design and sample	Parameters or spatial target	Principal finding	Relationship to the present study
Vilares-Morgado et al. (2024)	Longitudinal CXL outcomes; 41 eyes of 30 patients	Kmax, Kmean, astigmatism, minimum pachymetry, PRC, and Belin/Ambrosio D-index	Assessed multidimensional tomographic change for up to 48 months after transepithelial accelerated CXL.	Longitudinal and treatment-focused; did not measure the planar TP–Kmax distance.
Hammoud et al. (2025)	Prospective cross-sectional study; 95 eyes from 95 participants	Distance between the thinnest corneal point and focal biomechanical weakening	The thinnest-to-weakest-point distance was greater in subclinical and manifest KC than in controls.	Spatially relevant, but the second landmark was biomechanical weakening rather than Kmax.
Villa-Collar et al. (2026)	Retrospective cross-sectional study; 455 keratoconic eyes	Keratometry, pachymetry, aberrations, and thinnest-point location across four severity systems	Agreement among anterior-surface severity classifications was generally limited.	Supports multidimensional characterization; did not analyse TP–Kmax distance or posterior-surface ABCD components.

CXL, corneal cross-linking; Kmax, maximum keratometry; PRC, posterior radius of curvature; TP, thinnest point; KC, keratoconus. No study in the comparison evaluated the identical planar TP–Kmax distance used in the present study.
